# Intravitreal Injection of IGFBP-3 Restores Normal Insulin Signaling in Diabetic Rat Retina

**DOI:** 10.1371/journal.pone.0093788

**Published:** 2014-04-02

**Authors:** Youde Jiang, Qiuhua Zhang, Jena J. Steinle

**Affiliations:** 1 Department of Ophthalmology, University of Tennessee Health Science Center, Memphis, Tennessee, United States of America; 2 Department of Anatomy & Neurobiology, University of Tennessee Health Science Center, Memphis, Tennessee, United States of America; 3 Department of Pharmaceutical Sciences, University of Tennessee Health Science Center, Memphis, Tennessee, United States of America; Children's Hospital Boston, United States of America

## Abstract

Diabetes-induced changes in growth factor binding protein 3 (IGFBP-3) and tumor necrosis factor alpha (TNFα) have been linked to decreased insulin receptor signaling in diabetic retinopathy. Our previous studies in retinas of diabetic rats have shown that Compound 49b, a novel β-adrenergic receptor agonist, prevented diabetic changes by increasing IGFBP-3 and decreasing TNFα, thus restoring insulin signaling and protection against diabetic retinopathy. The current study was designed to determine whether boosted expression of IGFBP-3 NB (a non-IGF-1 binding form of IGFBP-3) *alone* is sufficient to mimic the full actions of Compound 49b in protecting against diabetic retinopathy, as well as testing whether IGFBP-3 NB is linked to a restoration of normal insulin signal transduction. Two months after initiation of streptozotocin-induced diabetes, rats received a single intravitreal injection of IGFBP-3 NB plasmid in the right eye. Four days after injection, electroretinogram (ERG) analyses were performed prior to sacrifice. Whole retinal lysates from control, diabetic, diabetic + control plasmid, and diabetic+ IGFBP-3 NB were analyzed for IGFBP-3, TNFα, suppressor of cytokine signaling 3 (SOCS3), and insulin receptor signaling partners using Western blotting or ELISA. Data show that a single intraocular injection of IGFBP-3 NB in diabetic animals significantly reduced TNFα levels, concomitant with reductions in IRS-1^Ser307^, SOCS3, and pro-apoptotic markers, while restoring insulin receptor phosphorylation and increasing anti-apoptotic marker levels. These cellular changes were linked to restoration of retinal function. Our findings establish IGFBP-3 as a pivotal regulator of the insulin receptor/TNFα pathway and a potential therapeutic target for diabetic retinopathy.

## Introduction

Rates of diabetes are expected to increase significantly over the next several years due to increased obesity of the population. Diabetic retinopathy will affect approximately 20% of people diagnosed with diabetes. While progress has been made in identifying potential pathways involved in diabetic retinopathy, current treatments remain limited. Our own studies have shown that loss of sympathetic neurotransmission, specifically β-adrenergic receptor signaling, produced retinal changes similar to that observed in diabetic animal models [1]. Likewise, we demonstrated that loss of dopamine beta hydroxylase (a key enzyme that converts dopamine to norepinephrine) [2], β-1-adrenergic receptor signaling [3], or β-2-adrenergic receptor signaling [4] can all produce a phenotype similar to diabetic retinopathy, in the absence of changes in glucose levels. Based on these findings, we tested whether or not the adrenergic receptor agonist, isoproterenol, could restore normal β-adrenergic receptor signaling in the eye and thus prevent retinal damage associated with diabetes using the streptozotocin-induced diabetes rat model. Results showed that as predicted, isoproterenol treatment prevented retinal damage; however, the treatment also caused unacceptable side effects in the heart [5]. To avoid these cardiovascular changes, we synthesized a novel β-adrenergic receptor agonist, Compound 49b, which selectively prevents both vascular and neuronal changes associated diabetes in the retina but has little effect on the heart [6].

The discovery of Compound 49b has not only provided an important potential treatment strategy for diabetic retinopathy, it also has provided a useful research tool for further analysis of pathways that alter diabetes-induced changes in retina. Our continuing studies of Compound 49b in the streptozotocin-induced diabetic rat model *in vivo* and in identified retinal cell types *in vitro* indicate that its likely mechanism of action is through increasing insulin-like growth factor binding protein 3 (IGFBP-3) levels, while decreasing tumor necrosis factor alpha (TNFα levels) [6]. Our data suggest that the effect of Compound 49b on IGFBP-3 does not involve interactions with the insulin-like growth factor, IGF-1, but rather independent actions of IGFBP-3 acting through the IGFBP-3 receptor [7], [8]. We have subsequently demonstrated that Compound 49b regulates IGFBP-3 through DNA-PK to prevent apoptosis of retinal endothelial cells [9]. Additionally, we have shown that IGFBP-3 regulates retinal endothelial cell apoptosis through binding to its receptor (serine/threonine kinase type V TGF-beta receptor (TGF-β RV, termed LRP1)[10]. Based upon our data with Compound 49b, we hypothesized that IGFBP-3 is upstream of TNFα, such that maintenance/restoration of IGFBP-3 actions would prevent TNFα's inhibition of insulin signaling.

Our findings of increased TNFα and SOCS3 in retinal endothelial and Müller cells under high glucose conditions agree well with findings in other cell types [11], [12], including adipocytes and smooth muscle cells, suggesting that these retinal cells may also undergo a form of insulin resistance. In normal insulin signal transduction, autophosphorylation of the insulin receptor on tyrosine 1150/1151 leads to activation of IRS-1 or IRS-2, which phosphorylate Akt, a potent anti-apoptotic factor, thus preventing apoptosis of cells. We have shown that TNFα blocks normal insulin signal transduction in both retinal endothelial and Müller cells [13], [14]. Under high glucose conditions, TNFα increases phosphorylation of insulin receptor substrate 1 (IRS-1) on serine 307[15], [16], thus inhibiting the ability of IRS-1 to activate Akt to prevent apoptosis. TNFα can also regulate insulin signal transduction through increasing levels of suppressor of cytokine signaling 3 (SOCS3) [17]. SOCS3 is reported to inhibit insulin signaling by multiple potential mechanisms, including increased phosphorylation of insulin receptor on tyrosine 960 (IR^Tyr960^) and thus inhibiting the complex formation between insulin receptor and IRS-1 [18]. SOCS3 also can promote ubiquitinization of IRS-1, thus blocking insulin signal transduction [19]. Since we have reported that IGFBP-3 and TNFα work antagonistically in retinal endothelial cells [20], we questioned whether IGFBP-3 may act upstream to inhibit TNFα and thereby blunt glucose-dependent effects of TNFα on suppression of insulin signal transduction. Our results confirm this hypothesis and demonstrate that treatment of diabetic rats with IGFBP-3 plasmid that does not bind IGF-1 (NB) restores normal insulin transduction via a decrease in TNFα, SOCS3, and phosphorylation of IRS-1^Ser307^ and IR^Tyr960^.

## Methods

### Animals

All animal experiments were approved by the University of Tennessee Health Science Center Institutional Animal Care and Use Committee (Protocol #1992) and follow the Association for Research in Vision Research guidelines. Thirty male Lewis rats (Charles River, Wilmington, MA) were made diabetic by a single injection of streptozotocin (60 mg/kg, IP, Fisher Scientific, Pittsburgh, PA) at 8 weeks of age. Diabetic status was verified by glucose strip measurement and considered diabetic if glucose was >250 mg/dl. Ten control rats were also used. Glucose and body weight were measured weekly. No insulin was administered. Four groups of rats were used for these experiments: control, diabetic only, diabetic+control expression plasmid with CMV promoter (CMV control vector) and diabetic+IGFBP-3 NB. We injected the right eye with the left eye left as the contralateral control. For all data, we present the control, diabetic and diabetic+treated eye and diabetic+contralateral eye to provide six data groups for analyses. We chose the experimental design to inject the right eye of diabetic animals, leaving the left eye as an intra-animal control, rather than treating one eye with IGFBP-3 NB or control plasmid and the other eye of the same animal with the control vector to allow assessment of whether IGFBP-3 NB was detected in the contralateral eye. This is key for future work on whether IGFBP-3 can be used as a therapeutic for diabetic retinopathy. All animals were made diabetic at the same time. While we may have performed a needle poke as a sham to the contralateral eye, the data suggest that this would not have altered our results, as TNFα levels were not reduced by IGFBP-3, rather than increased as would be expected by needle poke. Future work will include this additional control. Table 1 shows body weight and blood glucose measurements at sacrifice.

**Table 1:** Measurements of body weight, blood glucose and intraocular pressure (IOP) in control (Ctrl), diabetic (diabetic), diabetic+IGFBP-3 intravitreal injection (Diab+Bp3), diabetic+IGFBP3 contralateral eye (Diab+BP3 Contra), diabetic+CMV intravitreal injection (Diab+CMV), and diabetic+CMV contralateral eye (Diab+CMV Contra).

Groups	N	Body Weight (g)	Glucose (mg/dl)	IOP
Ctrl	10	376.7±15.5	112.7±12.3	12±3.5
Diabetic	10	203.3±23.5*	524.6±43.2*	11±4.5
Diab+BP3	10	209.6±18.3*	535.8±67.2*	10±3.7
Diab+BP3 Contra	10	209.6±18.3*	535.8±67.2*	10±4.4
Diab+CMV	9	205.1±13.3*	530.4±58.4*	12±3.1
Diab+CMV Contra	9	205.1±13.3*	530.4±58.4*	11±4.8

*P<0.05 vs. control.

After 2 months of diabetes, 10 rats received a single intravitreal injection of IGFBP-3 NB plasmid (1 ug/ul in 1 ul volume), a plasmid of IGFBP-3 that does not bind IGF-1 [10] into the right eye. In 10 other diabetic animals, the right eye was treated with a control plasmid. Four days after injection of IGFBP-3 NB or control plasmid, electroretinogram analyses were performed on each eye, followed by sacrifice and isolation of retina.

### Electroretinogram (ERG)

ERG analyses were carried out to evaluate the changes in the electrical activity of the retina between the right and left eye of control, diabetic, diabetic+CMV, and diabetic+IGFBP-3 NB animals [4], [20]. Briefly, rats were dark-adapted overnight and ERG responses were recorded from both eyes together using platinum wire corneal electrodes, forehead reference electrode, and ground electrode in the tail. Pupils were dilated using 1% tropicamide solution (Alcon). Methylcellulose (Celluvise; Allergan, Irvine, CA) drops were applied as well to maintain a good electrical connection and body temperature was maintained at 37°C by a water-based heating pad. ERG waveforms were recorded with a bandwidth of 0.3–500 Hz and samples at 2 kHz by a digital acquisition system and were analyzed a custom-built program (MatLab). Statistics was done on the mean ±SD amplitudes of the a- and b- wave of each treatment group, including oscillatory potentials. Comparisons were made between the right (OD) and left eyes (OS).

Intraocular pressure (IOP) was measured monthly using a tonometer (TonoLab, Colonial Medical Supply, Franconia, NH). Briefly, the tip of the probe of the tonometer was placed at the cornea of the eye. During measurements, the tip of the probe hit the cornea six times and gave the IOP reading of that eye.

### Western Blotting

After appropriate treatments and rinsing with cold phosphate-buffered saline, retinal lysates were placed into lysis buffer containing the protease and phosphatase inhibitors. Equal amounts of protein from the retinal lysates were separated on the pre-cast tris-glycine gel (Invitrogen, Carlsbad, CA), blotted onto a nitrocellulose membrane. After blocking in TBST (10 mM Tris-HCl buffer, pH 8.0, 150 mM NaCl, 0.1% Tween 20) and 5% (w/v) BSA, the membrane was treated with appropriate primary antibodies followed by incubation with secondary antibodies labeled with horseradish peroxidase. Antigen-antibody complexes were detected by chemilluminescence reagent kit (Thermo Scientific, Pittsburgh, PA). Primary antibodies used were phosphorylated Akt (Serine 473), Akt, total IRS-1, phosphorylated IRS-1^Ser307^, total insulin receptor, phosphorylated insulin receptor on Tyr 1150/1151, Bax, Bcl-xL, and SOCS3 (all purchased from Cell Signaling, Danvers, MA), beta actin (Santa Cruz), and insulin receptor (Tyr 960, Cell Applications).

### ELISA

A cleaved caspase 3 ELISA (Cell Signaling, Danvers, MA) was used to measure levels of the active apoptotic marker in the retinal lysates, with 50 ug loaded to allow for analyses using optical density measurements as no standard curve is provided. TNFα protein concentrations were measured using a rat TNFα ELISA with 50 ug used for loading. (ENER3TNFA, ThermoFisher, Pittsburgh, PA). An IGFBP-3 ELISA was done to verify that injections increased IGFBP-3 levels in the retina (BioVendor, Asheville, NC).

### Statistics

A 1-way ANOVA with Bonferroni's post test was used for statistical analyses. Electroretinogram analyses are presented as mean ± SD. All Western blot densitometric measurements and ELISA analyses are presented as mean ± SEM. For Western blots of phosphorylated proteins, the ratio of phosphorylated to total protein is presented. If the protein is not phosphorylated, the ratio of actin is presented. A representative blot is provided.

## Results

### Intravitreal IGFBP-3 NB reduced TNFα levels in the treated eye

**Figure 1:**
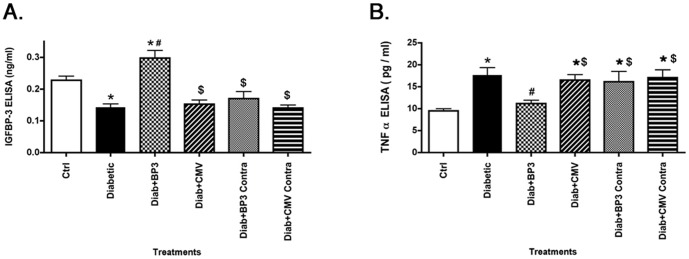
Intravitreal injection of IGFBP-3 plasmid increases IGFBP-3, while reducing TNFα levels. For all studies, groups are control (ctrl), diabetic (Diabetic), Diabetic +IGFBP-3 NB (Diab +BP3), Diabetic +CMV (Diab+CMV), Diabetic+IGFBP-3 NB left eye (Diab+BP3 contra) and Diabetic+CMV left eye (Diab+CMV Contra). Panel A shows ELISA results for IGFBP-3 demonstrating that the plasmid injection did significantly increase IGFBP-3 levels. Panel B demonstrates that intravitreal injection of IGFBP-3 NB significantly reduced TNFα levels. *P<0.05 vs ctrl. #P<0.05 vs. Diabetic only, $P<0.05 vs. Diab+BP3. N = 5 for all groups Data are mean ± SEM.

Intravitreal injections of IGFBP-3 NB into the right eye of rats two months after the onset of diabetes significantly increased IGFBP-3 levels in the treated eye (Figure 1A); no changes were observed in the untreated (left) eye. This experiment verified that IGFBP-3 NB plasmid was effective in increasing IGFBP-3 NB but that it did not cross into the contralateral eye, nor did the control plasmid have any effect on IGFBP-3 levels. Two months of diabetes significantly increased retinal TNFα levels (Figure 1B). When IGFBP-3 NB was injected into the right eye of 2 month diabetic rats, the treated eye had a significant reduction in TNFα levels compared to untreated diabetic animals (Figure 1B), demonstrating that IGFBP-3 can reduce TNFα levels, in verification of our previous reports [20]. Additionally, we found that injection of IGFBP-3 plasmid significantly reduced TNFα compared to the contralateral eye injected with empty vector.

### IGFBP-3 NB increased insulin receptor phosphorylation and reduced IRS-1^Ser307^ in diabetic rat retina

**Figure 2:**
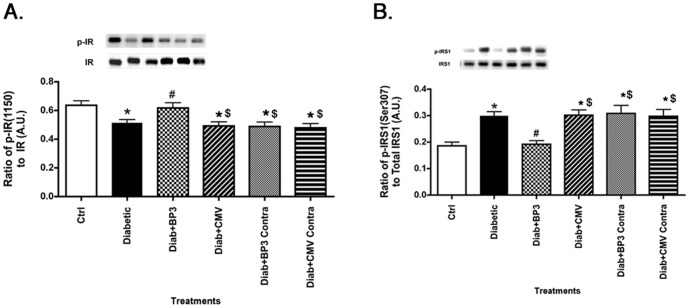
IGFBP-3 NB restores insulin receptor phosphorylation, while reducing IRS-1^Ser307^ phosphorylation. For all studies, groups are control (ctrl), diabetic (Diabetic), Diabetic +IGFBP-3 NB (Diab +BP3), Diabetic +CMV (Diab+CMV), Diabetic+IGFBP-3 NB left eye (Diab+BP3 contra) and Diabetic+CMV left eye (Diab+CMV Contra). Panel A presents Western blot results for the ratio of phosphorylated insulin receptor tyrosine 1150/1151 to total insulin receptor. Panel B shows the ratio of phosphorylated IRS-1 on serine 307 to total IRS-1. IRS-1^Ser307^ is inhibitory to insulin signal transduction. *P<0.05 vs ctrl. #P<0.05 vs. Diabetic only, $P<0.05 vs. Diab+BP3. N = 5 for all groups. Data are mean ± SEM.

We have previously reported that high glucose reduces insulin receptor phosphorylation on tyrosine 1150/1151 in retinal Müller cells [14]. Additionally, we have reported that high glucose increases TNFα, leading to increased phosphorylation of IRS-1^Ser307^ in retinal Müller and endothelial cells, which in turn is inhibitory to insulin signal transduction [13], [21]. Since IGFBP-3 NB reduced TNFα levels, we wanted to investigate whether IGFBP-3 NB injections could restore normal insulin signal transduction in diabetic rats. Data show that diabetes decreases insulin receptor phosphorylation (Figure 2A), while increasing IRS-1^Ser307^ phosphorylation (Figure 2B). Injection of IGFBP-3 NB restored insulin receptor phosphorylation to control levels (Figure 2A), and significantly reduced IRS-1^Ser307^ (Figure 2B). Injection of IGFBP-3 NB was significantly different than injection of empty vector for both insulin receptor and IRS-1^Ser307^.

### IGFBP-3 NB reduces SOCS3 levels, leading to decreased phosphorylation of IR^Tyr960^

**Figure 3:**
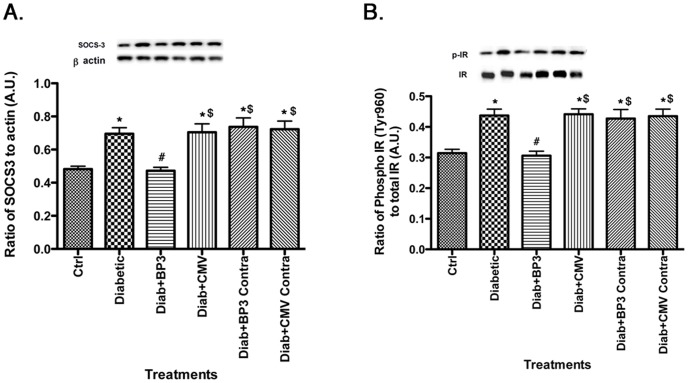
SOCS3 and insulin receptor phosphorylation on tyrosine 960 are reduced by IGFBP-3 NB injection. For all studies, groups are control (ctrl), diabetic (Diabetic), Diabetic +IGFBP-3 NB (Diab +BP3), Diabetic +CMV (Diab+CMV), Diabetic+IGFBP-3 NB left eye (Diab+BP3 contra) and Diabetic+CMV left eye (Diab+CMV Contra). Panel A shows that SOCS3 levels are decreased after IGFBP-3, measured by Western blotting. Panel B shows that IR^Tyr960^ phosphorylation is reduced, which follows the SOCS3 data since IR^Tyr960^ is activated by SOCS3. *P<0.05 vs ctrl. #P<0.05 vs. Diabetic only, $P<0.05 vs. Diab+BP3. N = 5 for all groups. Data are mean ± SEM.

We have previously reported that TNFα can regulate SOCS3 levels, leading to increased phosphorylation of IR^Tyr960^ in retinal endothelial cells [13]. While we have shown that IGFBP-3 can regulate TNFα, we have not previously investigated whether IGFBP-3 leads to an alteration in levels of TNFα's downstream target, SOCS3. Figure 3A demonstrates that diabetes increases SOCS3 levels, which is blocked in diabetic animals receiving the intravitreal injection of IGFBP-3 NB. Accordingly, IR^Tyr960^ phosphorylation is reduced in IGFBP-3 NB treated animals compared to untreated diabetic rats (Figure 3B). IGFBP-3 NB injection significantly decreased SOCS3 and IR^Tyr960^ when compared to injection of empty vector. These data further support that IGFBP-3, acting through the TNFα, pathway, can regulate insulin signal transduction.

### Apoptotic markers are reduced after IGFBP-3 NB injection

**Figure 4:**
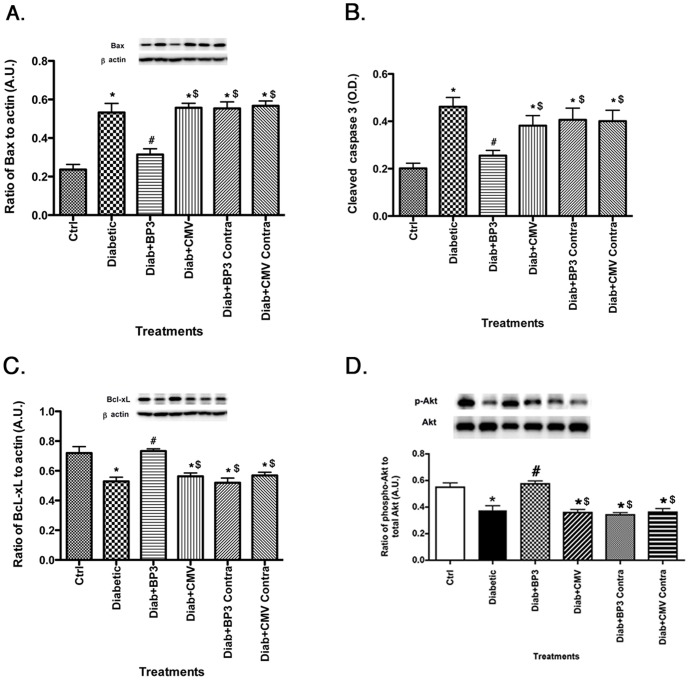
Pro-apoptotic markers are decreased after IGFBP-3 NB injections. For all studies, groups are control (ctrl), diabetic (Diabetic), Diabetic +IGFBP-3 NB (Diab +BP3), Diabetic +CMV (Diab+CMV), Diabetic+IGFBP-3 NB left eye (Diab+BP3 contra) and Diabetic+CMV left eye (Diab+CMV Contra). Panels A&B show that Bax (A) and cleaved caspase 3 (B) levels are all decreased after the IGFBP-3 NB intravitreal injections. Panels C&D show that Bcl-xL (C) and Akt (D) are increased, demonstrating that IGFBP-3 promotes survival of retinal cells during diabetes. *P<0.05 vs ctrl. #P<0.05 vs. Diabetic only, $P<0.05 vs. Diab+BP3. N = 5 for all groups. Data are mean ± SEM.

Since one key aspect of insulin signaling is activation of anti-apoptotic pathways, we measured a number of key apoptotic proteins [Bax (A), cleaved caspase 3 (B)] and anti-apoptotic [Bcl-xL (C), Akt (D)] in the retina of control, diabetic, diabetic+CMV, and diabetic+IGFBP-3 NB animals. Diabetes significantly increased apoptotic markers (top panels) and reduced anti-apoptotic proteins (bottom panels, Figure 4). In all cases, IGFBP-3 NB reversed these changes by reducing apoptotic proteins and increasing anti-apoptotic proteins. IGFBP-3 NB injection increased anti-apoptotic proteins and decreased pro-apoptotic proteins when compared to injection alone.

### IGFBP-3 NB injection improves ERG amplitudes compared to untreated eye

**Figure 5:**
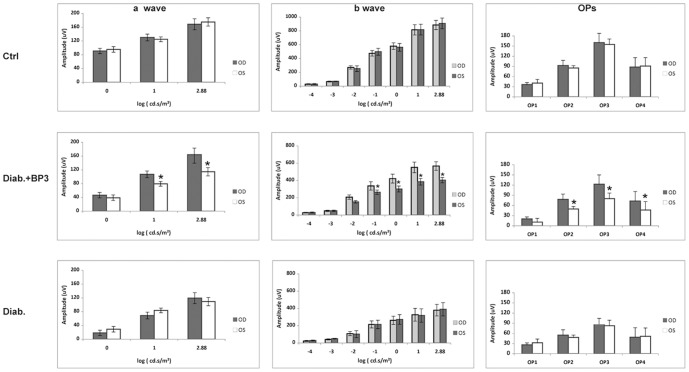
IGFPB-3 injection improves ERG amplitudes in treated eye. Electroretinogram results for control, diabetic, and diabetic +IGFBP-3 treated animals. Left panels are A-wave, middle panels are B-wave and right panels show the oscillatory potentials. Data is shown for the right eye (OD) versus the left eye (OS). No changes are noted in the control or diabetic animals between ERG amplitudes. In the diabetic +IGFBP-3 treated group, the right eye (treated) has significantly higher A-wave and B-wave amplitudes that the untreated (OS) eye. N = 5 for all groups. Data are mean ± SD.

There are no differences in the ERG amplitudes between the right (OD) and left (OS) eyes in control or diabetic animals after 2 months of diabetes. In the diabetic+IGFBP-3 NB group, intravitreal injection of IGFBP-3 NB significantly improved the ERG amplitudes for the a-wave, b-wave and oscillatory potentials after 4 days in the treated eye (OD) compared to untreated eye (OS, Figure 5). Injection of empty vector did not alter ERG amplitudes compared to control or diabetes alone (data not shown). Importantly, these results suggest that IGFBP-3 may have potent effects on the retinal function, which may be linked to restoration of normal insulin signal transduction and decreased apoptotic proteins.

## Discussion

We recently demonstrated that IGFBP-3 knockout mice have neuronal characteristics of diabetes and that IGFBP-3 and TNFα have antagonistic actions on apoptosis in retinal endothelial cells [20]. Additionally, we have reported that Compound 49b is protective to the diabetic retina through increased IGFBP-3 levels [6]. Therefore, the next question was whether restoration of IGFBP-3 alone was sufficient to protect the retina, and whether the actions of IGFBP-3 involve reduction of TNFα levels. Data in Figure 1 demonstrate that intravitreal injections of IGFPB-3 NB does significantly increase IGFBP-3 in retinal lysates, which in turn was associated with decreased TNFα levels in the treated eye. Additionally, the unilateral injection did not affect the contralateral eye, as no changes were observed in the untreated eye for any of the analyses.

**Figure 6:**
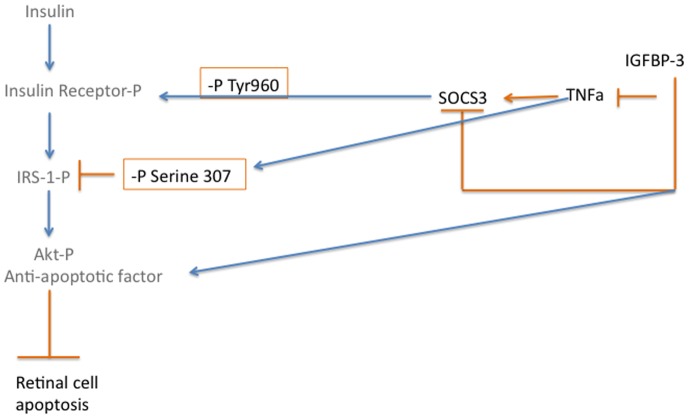
Schematic of the regulation of insulin signal transduction by IGFBP-3 and TNFα.

One of the potential complications of diabetes is insulin resistance, particularly in type 2 diabetic patients. We have recently reported that Compound 49b can reduce TNFα, IRS-1^Ser307^, and SOCS3 levels to reduce apoptosis of retinal Müller cells; these effects did not involve IGFBP-3 [22]. Additionally, we demonstrated in retinal endothelial cells that TNFα activation of SOCS3 and IRS-1^Ser307^ leads to decreased insulin signal transduction, which likely underlies insulin resistance. In the work reported here, we wanted to ascertain whether restoration of IGFBP-3 levels in whole retina could prevent the reduction in insulin resistance observed in retinal cells cultured in high glucose. Despite IGFBP-3 having limited actions in retinal Müller cells in culture [22], intravitreal injection of IGFBP-3 NB was able to significantly increase phosphorylation of insulin receptor in total retinal lysates, leading to activation of insulin signaling. Additionally, IGFBP-3 NB reduced IRS-1^Ser307^ and IR^Tyr960^; these two sites are inhibitory to insulin signal transduction. IGFBP-3 actions on IR^Tyr960^ are likely through the reduction in SOCS3 levels. While we did not use direct inhibition of SOCS3 in this study, we have previously demonstrated that TNFα can activate SOCS3 in retinal endothelial cells, leading to increased apoptosis [13]. When SOCS3 siRNA was used, phosphorylation of IR^Tyr960^ and apoptosis were reduced, suggesting a direct interaction between reduction of TNFα and SOCS3, leading to decreased apoptosis. We hypothesize that the ability of IGFBP-3 to maintain proper insulin signal transduction is largely due to its reduction of TNFα. Thus IGFBP-3 would be upstream to TNFα in the pathway regulating insulin signal transduction (see diagram of proposed pathways, Fig 6). In support of this notion, studies using other organs report a strong association between TNFα and dysfunctional insulin signal transduction through activation of SOCS3 [23], [24] or phosphorylation of IRS-1^Ser307^ [16].

A key outcome of insulin receptor signal transduction is activation of the anti-apoptotic factor, Akt, and decreased apoptosis. We have previously reported that Compound 49b restored IGFBP-3 levels in diabetic animals, reduced apoptosis in the retina, and increased phosphorylation of Akt [6]. We now show that increasing IGFBP-3 through an intravitreal injection of IGFBP-3 NB can directly reduce apoptotic markers, while increasing two anti-apoptotic markers, including Akt and Bcl-xL. While this study did not measure apoptosis in particular cell types, we believe that the reduced apoptosis is likely occurring in retinal endothelial cells as we have previously reported that IGFBP-3 is key to retinal endothelial cell survival [20]. This reduction in apoptosis was associated with improvement of the ERG in the treated eye. It is unclear whether the improvement was a direct or indirect effect, since the ERG improvement occurred so rapidly after treatment. Additionally, since ERG does not directly test retinal degeneration, further work would be required to validate the retinal changes resulting in altered ERG responses. Nonetheless, the finding warrants further study and highlights the potential importance of IGFBP-3 in protection/maintenance of retinal function. While we cannot rule out actions of IGF-1 receptor signaling in the observed changes, the use of the IGFBP-3 NB strongly suggests that IGFBP-3 is acting independent of changes in IGF-1.

In conclusion, our data demonstrate that an intravitreal injection of IGFBP3 NB is able to reduce TNFα levels in diabetic rats. This reduction in TNFα is associated with reduced SOCS3, IRS-1^Ser307^, and IR^Tyr960^ levels in retinal lysates from the treated eye. IGFBP-3 NB injected eyes had reduced apoptosis markers, likely associated with maintenance of insulin receptor signaling, indicated by phosphorylation of the insulin receptor. Additionally, IGFBP-3 NB treatment of diabetic rats improved the ERG measured at 4 days after intravitreal injection. These findings support the hypothesis that insulin signaling pathways in retina play a key role in maintaining retinal function and that diabetic insult targets these pathways to produce the diabetic retinopathy phenotype.
